# Proteome Profiling of Wheat Shoots from Different Cultivars

**DOI:** 10.3389/fpls.2017.00332

**Published:** 2017-03-13

**Authors:** Lam Dai Vu, Inge Verstraeten, Elisabeth Stes, Michiel Van Bel, Frederik Coppens, Kris Gevaert, Ive De Smet

**Affiliations:** ^1^Department of Plant Biotechnology and Bioinformatics, Ghent UniversityGhent, Belgium; ^2^Center for Plant Systems Biology, VIBGhent, Belgium; ^3^Medical Biotechnology Center, VIBGhent, Belgium; ^4^Department of Biochemistry, Ghent UniversityGhent, Belgium

**Keywords:** wheat, proteome, phosphoproteome, mass spectrometry, leaf

## Abstract

Wheat is a cereal grain and one of the world’s major food crops. Recent advances in wheat genome sequencing are by now facilitating its genomic and proteomic analyses. However, little is known about possible differences in total protein levels of hexaploid versus tetraploid wheat cultivars, and also knowledge of phosphorylated wheat proteins is still limited. Here, we performed a detailed analysis of the proteome of seedling leaves from two hexaploid wheat cultivars (*Triticum aestivum* L. Pavon 76 and USU-Apogee) and one tetraploid wheat (*T. turgidum* ssp. *durum* cv. Senatore Cappelli). Our shotgun proteomics data revealed that, whereas we observed some significant differences, overall a high similarity between hexaploid and tetraploid varieties with respect to protein abundance was observed. In addition, already at the seedling stage, a small set of proteins was differential between the small (USU-Apogee) and larger hexaploid wheat cultivars (Pavon 76), which could potentially act as growth predictors. Finally, the phosphosites identified in this study can be retrieved from the in-house developed plant PTM-Viewer (bioinformatics.psb.ugent.be/webtools/ptm_viewer/), making this the first searchable repository for phosphorylated wheat proteins. This paves the way for further in depth, quantitative (phospho)proteome-wide differential analyses upon a specific trigger or environmental change.

## Introduction

The widespread cultivation since centuries has firmly established wheat (*Triticum* ssp.) as one of the most important human food sources as well as livestock feed, especially in temperate climate areas. Currently, worldwide wheat agriculture is overwhelmingly composed of the common wheat (*Triticum aestivum*) which accounts for 95% of wheat production, whereas most of the remaining 5% is attributed to durum wheat (*T. turgidum* ssp. *durum*) (Shewry, 2009; Peng et al., 2011). The genome of domestic wheat consists of DNA from two progenitor species in the case of *T. turgidum* (AABB), which gives rise to the hexaploid *T. aestivum* (AABBDD) by hybridization with the diploid grass *Aegilops tauschii* (Shewry, 2009). The crop has shown to be sensitive to a wide range of environmental stresses (Lobell et al., 2011; Juroszek and von Tiedemann, 2013; Ray et al., 2015). Despite its significance in agriculture, the complex polyploid nature and its large genome size have remained a challenge for the acquisition of the domestic wheat sequence, which in turn brings difficulties to high-throughput, omics-type experiments. However, recent advances in wheat genome analysis^1^ now provide a starting point for detailed and global analyses.

The unique character of protein dynamics, defined by protein–protein interactions and post-translational modifications (PTMs) of various amino acids, makes proteins the key controllers or regulators in a vast number of cellular processes. It is therefore important to understand plant growth, development and responses to the environment on the protein level. The recent years have witnessed a steady increase in the application of mass spectrometry (MS)-based proteomics in wheat. Such studies were mostly aiming at revealing key pathways and regulators involved in developmental processes or in stress response (Komatsu et al., 2014; Kosová et al., 2014; Hu et al., 2015). Recent advances in bioinformatics have enabled label-free protein quantification (LFQ), which circumvents metabolic labeling of plant proteomes, such as stable isotopic labeling *in planta* (SILIP), which uses growth media enriched in ^14^N or ^15^N-coded salts (Schaff et al., 2008; Guo and Li, 2011; Arsova et al., 2012), or post-metabolic labeling of proteins and peptides (e.g., using isobaric tags for relative and absolute quantitation (iTRAQ) or tandem mass tags (TMT)). These advances further allow simultaneous analysis of higher numbers of samples (Schulze and Usadel, 2010; Nahnsen et al., 2013; Olsen and Mann, 2013). LFQ is not limited to shotgun proteomics experiments, but enabled quantifying PTMs in plant proteomes, thus allowing integrative in-depth analyses of protein levels and their modifications (Li et al., 2015; Silva-Sanchez et al., 2015; Wu et al., 2015). This approach of simultaneously analysing the phosphoproteome and the proteome was demonstrated in several studies (Bonhomme et al., 2012; Facette et al., 2013; Zhang et al., 2013; Roitinger et al., 2015; Vu et al., 2016), whereas the actual proteome dataset was rather rarely used to correct changes in phosphopeptide levels based on changes in the overall protein levels (Roitinger et al., 2015; Vu et al., 2016).

Plant (phospho)proteome databases have accumulated a large amount of information on the dicot model plant *Arabidopsis thaliana* and the cereal crop plant *Oryza sativa*, largely due to their completely sequenced genomes, and, to a much lesser extent, also for several other species (Durek et al., 2009; Wienkoop et al., 2012; Yao et al., 2014; Vu et al., 2016). However, while (phospho)proteomics in wheat is emerging (Komatsu et al., 2014; Kosová et al., 2014; Zhang et al., 2014a,b; Chateigner-Boutin et al., 2015; Chen et al., 2016), it is still in its infancy and information on the wheat proteome and phosphoproteome remains limited. Recently, however, an important step forward came from an extensive *T. aestivum* proteomic map of different organs and developmental stages (Duncan et al., 2016).

To gain insights into the proteome of tetraploid versus hexaploid wheat cultivars, and potentially into the contribution of the genome to protein abundance, and to extend our knowledge of phosphorylated wheat proteins, we applied our recently developed (phospho)proteomics workflow (Vu et al., 2016). We report on the limited differences between the proteomes of the wheat selected cultivars, suggesting that candidate protein discovery for further characterization can be done in either of these. In addition, we aimed to assess the possibility of identifying protein-level growth predictors by comparing a small and a large wheat cultivar, at a seedling stage where they are largely similar. Finally, there is – to the best of our knowledge – no searchable plant database available that holds information about wheat phosphoproteomes and that can further be readily queried by the general research community. In order to accommodate the growing interest in crop PTM-proteomics we added the phosphorylated wheat proteins identified in this study to our previously initiated plant PTM Viewer^2^.

## Materials and Methods

### Wheat Plant Materials

Seeds of two common bread wheat cultivars (*T. aestivum*), the dwarf USU-Apogee and the semi-dwarf Pavon 76, and the durum wheat (*T. turgidum* ssp. *durum*) cultivar Senatore Cappelli were surface sterilized by washing with 70% ethanol, followed by immersion in 5% sodium hypochlorite for 30 min and were finally washed three times with MilliQ water. Seeds were submerged in water and stratified in the dark at 4°C for 7 days to synchronize the germination process. Next, seeds were put in plastic boxes containing half-strength Murashige and Skoog (1/2 MS) supplemented with 0.8% agar and seedlings were grown at 21°C and under constant white light (100 μE m^-2^ s^-1^ photosynthetically active radiation, supplied by cool-white fluorescent tungsten tubes, Osram) for 5 days. The shoots of seedlings from uniformly germinated seeds were collected and frozen in liquid nitrogen.

### Salt Treatment

Surface sterilized *T. aestivum* cv. USU-Apogee and Pavon 76 seeds were germinated on half strength MS medium containing 0.8% agar. Two days after germination, seedlings uniform in size were transferred to test tubes containing full strength MS medium with 30 g/L sucrose and 0.8% agar with or without 100 mM NaCl. Plant growth was evaluated 14 days after the transfer. Shoot length (top of the longest leave up to junction with primary root) was recorded as representable measures for plant growth in the presence/absence of salt.

### Protein Extraction and Trypsin Digestion

Protein extraction was performed on three biological replicate samples (leaf material from independent plants) per wheat cultivar. One gram of finely ground plant material was suspended in homogenization buffer containing 50 mM Tris-HCl buffer (pH 8), 30% sucrose, 5 mM EDTA, and 1 mM DTT in Milli-Q water, to which the appropriate amounts of the cOmplete^TM^ protease inhibitor mixture (Roche) and the PhosSTOP phosphatase inhibitor mixture (Roche) were added. The samples were sonicated on ice and centrifuged at 4°C for 15 min at 2500 × *g* to remove debris. Supernatants were collected and a methanol/chloroform precipitation was carried out by adding 3, 1, and 4 volumes of methanol, chloroform and water, respectively. Samples were centrifuged for 10 min at 5000 × *g* and the aqueous phase was removed. After addition of four volumes of methanol, the proteins were pelleted via centrifugation for 10 min at 2500 × *g*. Pellets were washed with 80% acetone and re-suspended in 6 M guanidinium hydrochloride in 50 mM triethylammonium bicarbonate (TEAB) buffer (pH 8). Alkylation of cysteines was carried out by adding a combination of tris(carboxyethyl)phosphine (TCEP, Pierce) and iodoacetamide (Sigma-Aldrich) to final concentrations of 15 mM and 30 mM, respectively, and the samples were incubated for 15 min at 30°C in the dark. Before digestion, the samples were buffer-exchanged to 50 mM TEAB buffer (pH 8) using Illustra NAP-10 columns (GE Healthcare Life Sciences). The protein concentration was measured using the Bio-Rad Protein Assay. One mg of protein material was digested with the MS grade trypsin/endoproteinase-Lys-C mix (Promega) overnight at 37°C at an enzyme-to-substrate ratio of 1:100 (w:w). The digest was acidified to pH ≤ 3 with trifluoroacetic acid (TFA) and desalted using SampliQ C18 SPE cartridges (Agilent) according to the manufacturer’s guidelines. For phosphopeptide enrichment, the desalted peptides were fully dried in a vacuum centrifuge and then re-suspended in 130 μl of loading solvent [80% (v/v) acetonitrile, 5% (v/v) TFA]. For shotgun proteome analysis, 30 μl was vacuum dried and re-dissolved in 30 μl of 2% (v/v) acetonitrile and 0.1% (v/v) TFA.

### Phosphopeptide Enrichment

For phosphopeptide enrichment, 100 μl of the re-suspended peptides was incubated with 1 mg MagReSyn^®^ Ti-IMAC microspheres for 20 min at room temperature. The microspheres were washed once with wash solvent 1 (80% acetonitrile, 1% TFA, 200 mM NaCl) and twice with wash solvent 2 (80% acetonitrile, 1% TFA). The bound phosphopeptides were eluted with three volumes (80 μl) of a 1% NH_4_OH solution, immediately followed by acidification to pH ≤ 3 using formic acid. Prior to MS analysis, the samples were vacuum dried and re-dissolved in 50 μl of 2% (v/v) acetonitrile and 0.1% (v/v) TFA.

### LC-MS/MS Analysis

Each sample was analyzed twice (i.e., in two technical replicates) via LC-MS/MS on an Ultimate 3000 RSLC nano LC (Thermo Fisher Scientific) in-line connected to a Q Exactive mass spectrometer (Thermo Fisher Scientific). The sample mixture was first loaded on a trapping column (made in-house, 100 μm internal diameter (I.D.) × 20 mm, 5 μm C18 Reprosil-HD beads, Dr. Maisch, Ammerbuch-Entringen, Germany). After flushing from the trapping column, the sample was loaded on an analytical column (made in-house, 75 μm I.D. × 150 mm, 3 μm C18 Reprosil-HD beads, Dr. Maisch). Peptides were loaded with loading solvent A (0.1% TFA in water) and separated with a linear gradient from 98% solvent A’ (0.1% formic acid in water) to 55% solvent B’ [0.1% formic acid in water/acetonitrile, 20/80 (v/v)] over 170 min at a flow rate of 300 nL/min. This was followed by a 5 min wash reaching 99% of solvent B’. The mass spectrometer was operated in data-dependent, positive ionization mode, automatically switching between MS and MS/MS acquisition for the 10 most abundant peaks in a given MS spectrum. The source voltage was 3.4 kV and the capillary temperature was set to 275°C. One MS1 scan (m/z 400–2000, AGC target 3 × 10^6^ ions, maximum ion injection time 80 ms) acquired at a resolution of 70000 (at 200 m/z) was followed by up to 10 tandem MS scans (resolution 17500 at 200 m/z) of the most intense ions fulfilling predefined selection criteria (AGC target 5 × 10^4^ ions, maximum ion injection time 60 ms, isolation window 2 Da, fixed first mass 140 m/z, spectrum data type: centroid, under fill ratio 2%, intensity threshold 1.7 × 10^4^, exclusion of unassigned, 1, 5–8, > 8 charged precursors, peptide match preferred, exclude isotopes on, dynamic exclusion time 20 s). The HCD collision energy was set to 25% Normalized Collision Energy and the polydimethylcyclosiloxane background ion at 445.120025 Da was used for internal calibration (lock mass).

### Database Searching

MS/MS spectra were searched against the UniProtKB *T. aestivum* database (100641 entries, version 08.2015) with the MaxQuant software (version 1.5.3.8) with a precursor mass tolerance set to 20 ppm for the first search (used for non-linear mass re-calibration) and to 4.5 ppm for the main search. Trypsin was selected as enzyme setting. Cleavages between lysine/arginine-proline residues and up to two missed cleavages were allowed. S-carbamidomethylation of cysteine residues was selected as a fixed modification and oxidation of methionine residues was selected as a variable modification. The false discovery rate for peptide and protein identifications was set to 1%, and the minimum peptide length was set to 7. The minimum score threshold for both modified and unmodified peptides was set to 30. The MaxLFQ algorithm allowing for label-free quantification (Cox et al., 2014) and the ‘matching between runs’ feature were enabled. For calculation of protein ratios, both unique and razor peptides (non-unique peptides that are assigned to a protein group with the largest number of identified peptides) were selected. It is important to note that it is a challenge to determine the exact contribution of each chromosome to the abundance of homeologous proteins due to the highly homologous sequences, because it usually requires unique peptides for protein identification and quantification to distinguish these homeologs. All MS proteomics data have been deposited to the ProteomeXchange Consortium via the PRIDE partner repository (Vizcaíno et al., 2014, 2016) with the dataset identifier PXD005437. Next, the ‘ProteinGroups’ output file generated by the MaxQuant search was loaded into the Perseus (version 1.5.2.6) data analysis software available in the MaxQuant package. Proteins that were quantified in at least three out of six replicates of at least one cultivar were retained. Log2 protein ratios of the protein LFQ intensities were centered by subtracting the median of the entire set of protein ratios per sample. Missing LFQ values were replaced by random, though low numbers that are drawn from the normal distribution, as such numbers point to rather low intensities of a protein or a phosphosite in the analyzed sample. A one-way ANOVA, with permutation-based FDR < 0.05 and 250 randomizations to correct for multiple-hypothesis testing, was carried out to test for differences between cultivars. Grouping of the technical replicates was preserved in randomizations for the ANOVA test. The statistically significant hits were then *Z*-scored and clustered into groups by a hierarchical clustering analysis using Pearson correlation metric and visualized using MultiExperiment Viewer (MeV, version 4.9.0).

### GO Categorization

Protein sequences of the wheat (phospho)proteome dataset were loaded in the PLAZA monocot 3.0 workbench (Proost et al., 2015) using the BLASTP function against the *O. sativa* ssp. Japonica database. The *E*-value threshold for BLASTP was set at < 1 × 10^-5^. GO categories of the BLASTP results were extracted from the functional annotation view and analyzed.

### Motif-X Analysis

The Motif-X algorithm (Chou and Schwartz, 2011) was used to extract significantly enriched amino acid motifs surrounding the identified phosphosites. The sequence window was limited to 13 amino acids and foreground peptides were pre-aligned with the phosphosite in the center of the sequence window. The *T. aestivum* UniProtKB proteome dataset was used as the background database. The occurrence threshold was set at the minimum of 20 peptides and the *P*-value threshold was set at < 10^-6^.

## Results and Discussion

### Experimental Set-up

For our analyses, we chose to focus on three wheat cultivars, namely the traditional tetraploid durum wheat (*T. turgidum* ssp. *durum* cv. Senatore Cappelli) and two hexaploid bread wheats (*T. aestivum* L. USU-Apogee and *T. aestivum* L. Pavon 76). *T. aestivum* L. Pavon 76 is a semi-dwarf wheat cultivar that is commonly used in breeding and crossing programs (Waines and Ehdaie, 2007). USU-Apogee is a common spring wheat cultivar developed for high yields in controlled environments (Bugbee et al., 1997; Doherty and Jones, 2011). Compared to other dwarf wheat variants, USU-Apogee shows advantageous features for laboratory-scale research such as a rapid growth rate and an early flowering time (23 days after seedling emergence in continuous light at a constant temperature of 25°C). In addition, this cultivar is resistant to leaf tip chlorosis that usually occurs in wheat under rapid growth conditions. The autumnal cultivar *T. turgidum* ssp. *durum* cv. Senatore Cappelli, an indigenous and ancient variety of durum wheat, was the only durum variant with brittle rachis used in the Italian breeding program (Watanabe, 2005).

**FIGURE 1:**
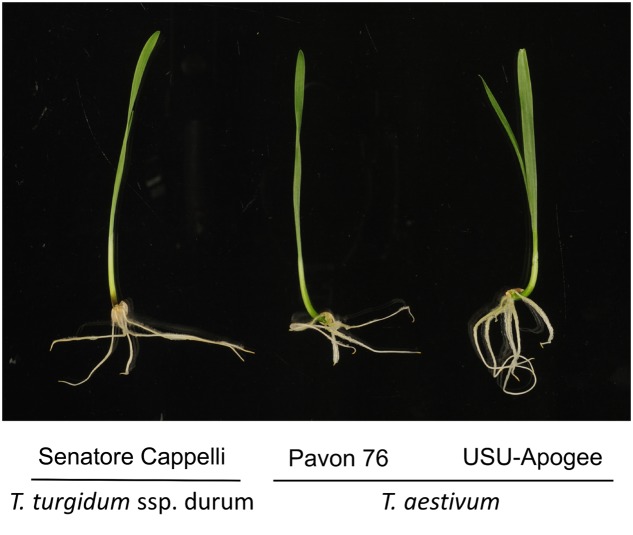
**Different wheat cultivars used in this study.** Seedlings are depicted at 5 days after germination.

**FIGURE 2:**
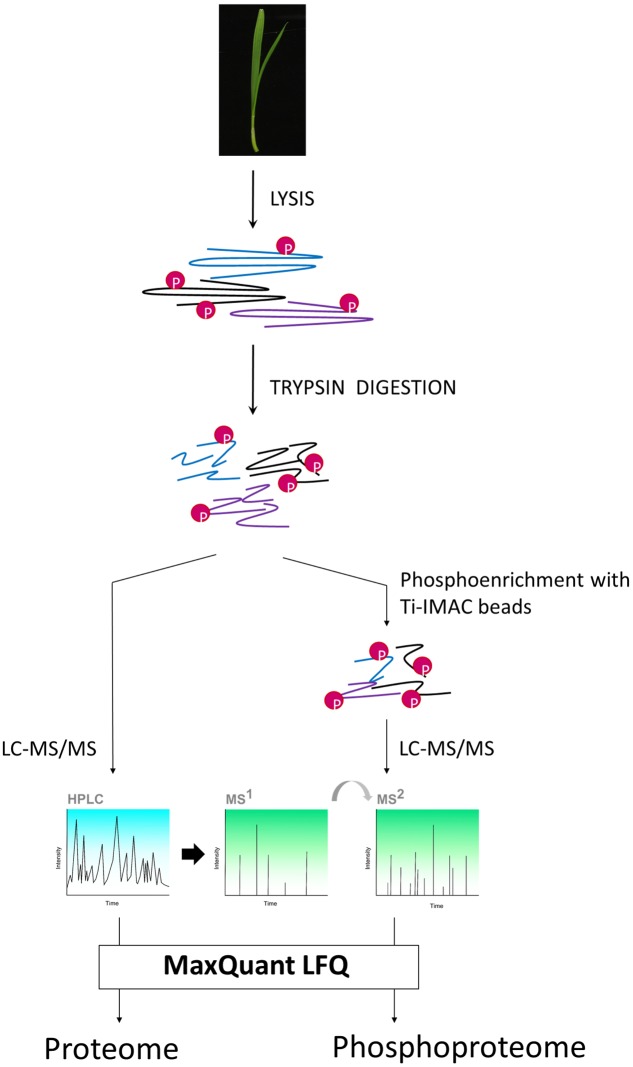
**(Phospho)proteomic workflow used in this study.** Wheat seedling leaves were sampled.

To compare the different variants, we opted to sample shoot (leaf) material from 7-day old seedlings (**Figure 1**). We then subjected this material to our recently developed (phospho)proteomics workflow (Vu et al., 2016) to map the proteome and the phosphoproteome in the leaves (**Figure 2**).

### Comparative Proteome Data Analysis

**FIGURE 3:**
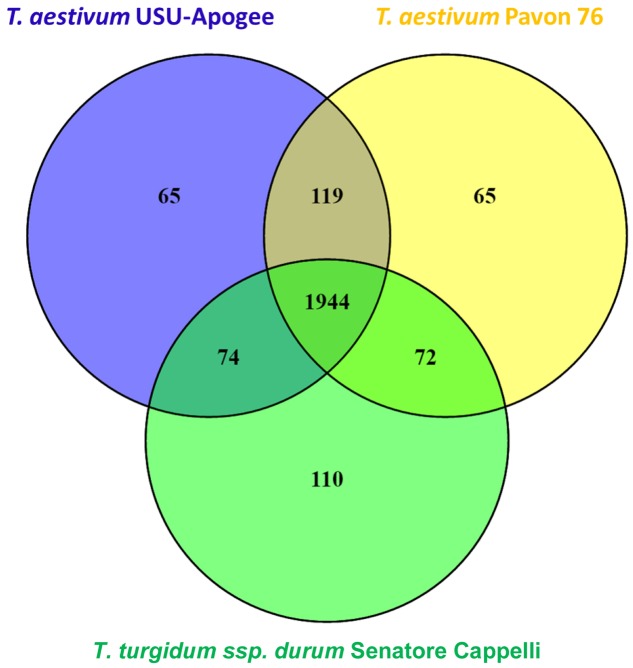
**Venn diagram representing the number of reproducibly quantified proteins for each wheat cultivar**.

Shotgun proteomics resulted in 282460 identified MS spectra that could be matched to 22578 peptides which were assigned to 4450 protein groups. Further filtering for proteins present in at least three out of six replicates in one of the cultivars and in at least one cultivar resulted in 2449 quantifiable proteins (Supplementary Table S1). A majority, namely 1944 of these reproducibly quantified proteins (79.3%), was found in all three cultivars (**Figure 3**).

**FIGURE 4:**
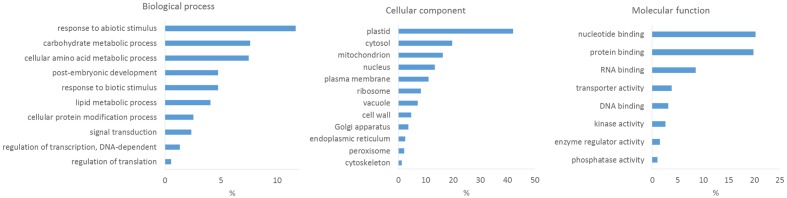
**GO categorization of quantifiable proteins in the three wheat cultivars**.

To obtain insights into the content of the wheat proteome dataset, we performed GO analyses using the PLAZA 3.0 workbench for monocots^3^, a versatile and freely accessible online tool for analysis and visualization of plant omics data (Proost et al., 2015). When functional annotation for an analyzed species is lacking, processing of proteome data usually involves searching for sequence homologs (via BLAST) of identified proteins in databases of related species with a sufficiently annotated genome to extract functional information. Whereas the genome sequence and annotation for other members of the *Triticum* genus does (at the moment) not exist in PLAZA 3.0, the database includes members of the Pooideae (true grass) family (such as *Brachypodium distachyon* and *Hordeum vulgare*) and also the cultivated rice (*O. sativa*) from the Oryzoideae subfamily in the Bambusoideae, Oryzoideae, and Pooideae clade. *O. sativa* ssp. japonica exhibits the highest numbers of GO terms as well as GO terms inferred from experimental evidences, and hence provided the most suitable database for the BLAST searches. In total, 2438 out of 2449 quantified wheat proteins could be matched to 1964 *O. sativa* proteins (*e*-value < 10^-5^). The lower number of *O. sativa* proteins that could be linked to the identified wheat proteins might be explained by the polyploidy of the domestic wheat, which often expresses homoeologous genes from two or three homoeoloci (Leach et al., 2014), or by the quality of the search database. The results revealed that proteins involved in biological processes, such as response to abiotic stimulus, amino acid metabolism and carbohydrate metabolism, are among the most present; while molecular functions were predicted to be involved in nucleotide binding, protein binding, RNA binding, transport activity, kinase activity, phosphatase activity and enzyme regulator activity. Among all cellular components, ‘plastid’ was predicted to have the largest number of identified and quantified proteins (**Figure 4**). Overall, we were able to gain insights into the proteome data, which is covering the expected protein groups.

**FIGURE 5:**
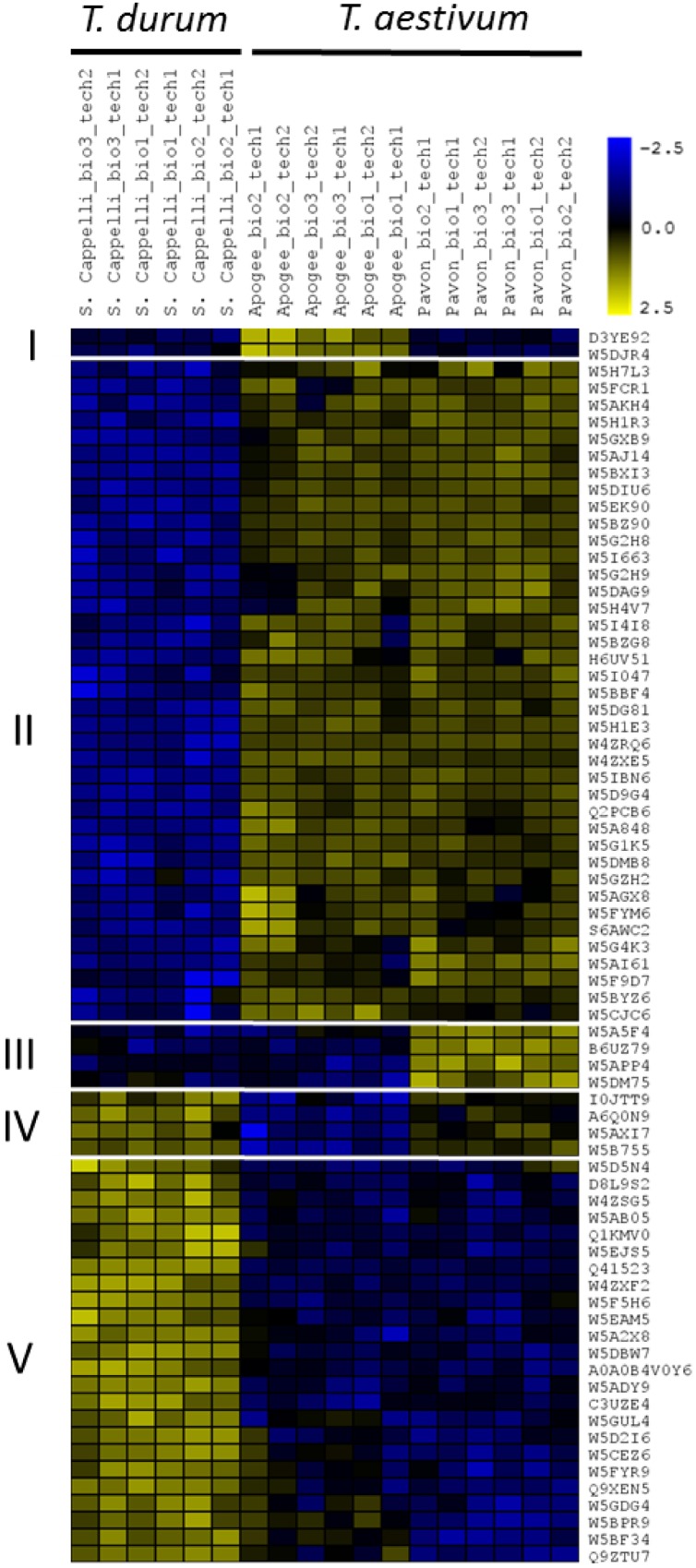
**Heat-map representing the proteins with significant different abundances in the technical replicates with the respective biological replicates of the wheat variants from the proteome analysis.** Imputed Log2-Intensities of the proteins were *Z*-scored for the clustering. Wheat IDs are shown and protein description taken from the orthologs in *Oryza sativa* can be found in Supplementary Table S2.

In total, 7150 missing intensities were replaced by values derived from the normal distribution. Subsequently, statistical multiple-sample testing (*p* < 0.05) on the imputed data revealed 73 proteins (out of 2449) that were significantly differentially abundant between the wheat cultivars (**Figure 5** and Supplementary Table S2). Hierarchical clustering of these 73 proteins based on their *Z*-scored log2 LFQ intensities showed a distinct difference in abundance of these proteins between *T. turgidum* ssp. *durum* cv. Senatore Cappelli samples and the hexaploid wheat samples. As expected, the two hexaploid cultivars exhibit a great similarity in abundance of these proteins with few exceptions. A mean distance threshold for clustering of 0.30 divided the 73 proteins into five terminal nodes representing five distinct sub-clusters holding proteins with differences in their abundance. The largest cluster (cluster II) contained 39 proteins that are less present in *T. turgidum* ssp. *durum* cv. Senatore Cappelli. Subsequent GO analysis of cluster II – using all the quantified proteins as background model (*p* ≤ 0.01) – showed a 40-fold enrichment of proteins involved in oxylipin biosynthesis (Supplementary Table S3). Oxylipins, for example jasmonic acid, are known as important signaling molecules during growth and especially in stress responses and innate immunity (Eckardt, 2008). Noticeably, four putative linoleate 9S-lipoxygenases (W5BZ90, W5BBF4, W5G4K3, and W5F9D7; Supplementary Table S2) that might play a role in stress-responsive oxylipin metabolism were more abundant in the hexaploid cultivars. In contrast, cluster V (24 proteins) contains proteins with higher abundance in the tetraploid wheat. Here, GO analysis on cluster V resulted in lignin biosynthesis as the only enriched specific term of biological processes, representing proteins with higher abundance in *T. turgidum* ssp. *durum* cv. Senatore Cappelli (Supplementary Table S3). Furthermore, three much smaller clusters, cluster I (two proteins), III (four proteins), and IV (four proteins), displayed proteins with different levels between the two hexaploid wheat cultivars. Possibly, these differences represent different response potential to environmental triggers, or underlie the growth potential of the two *T. aestivum* variants, making them useful growth predictors at an early seedling stage. For example, cluster I contained an HSP70-family DnaK chaperone (D3YE92) and the salt stress root protein (RS1; W5DJR4) that were more present in *T. aestivum* L. USU-Apogee compared to *T. aestivum* L. Pavon 76 (8.3- and 17.2-fold, respectively) and compared to *T. turgidum* ssp. *durum* cv. Senatore Cappelli (10.2- and 18.8-fold, respectively), possibly helping the plants to anticipate and/or survive stressful growth conditions (Wang et al., 2004). On the other hand, cluster IV contained two redox-active enzymes, an isoflavone reductase homolog (W5A5F4) and an quinone-oxidoreductase homolog (W5APP4) that were more abundant in *T. aestivum* L. Pavon 76 compared to *T. aestivum* L. USU-Apogee (3.4- and 5-fold, respectively) and compared to *T. turgidum* ssp. *durum* cv. Senatore Cappelli (4.7- and 3.8-fold, respectively). In this context, it has, for example, been shown in rice that the expression of the isoflavone reductase gene *OsIRL* is induced by oxidants (Kim et al., 2010). Hence, accumulation of isoflavone reductase proteins might increase oxidative stress tolerance in the cultivar. Overall, it seems thus tempting to speculate that the difference between the wheat cultivar proteomes might result from distinct developmental or physiological traits, reflected for instance in the different abundance of proteins involved in secondary metabolism, but this might also hint to differences in the resistance to environmental stresses of different wheat cultivars, especially between tetraploid and hexaploid wheat (Sairam et al., 2001; Yang et al., 2014; Li et al., 2017).

Some of the differences identified in this study might allow increasing our insights into genome interaction in protein expression. Although for some proteins large differences between tetraploid and hexaploid wheat varieties have been detected (Islam et al., 2003), our study seems to suggest that this cannot be generalized.

### Salt Stress Tolerance of USU-Apogee and Pavon 76 Seedlings

**FIGURE 6:**
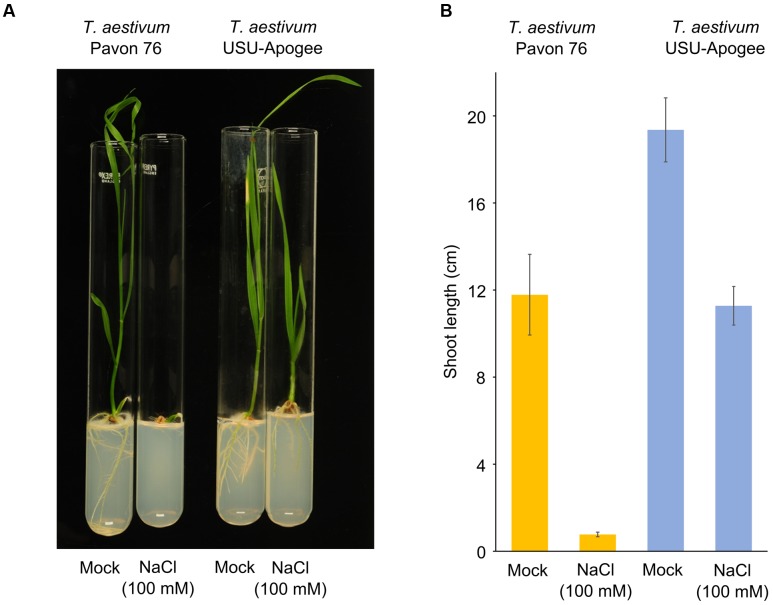
**Salt stress tolerance in *T. aestivum* Pavon 76 and USU-Apogee seedlings.** **(A)** Representative picture of 14-day-after-treatment seedlings of the cultivars under control and salt stress conditions. **(B)** Shoot length quantification of *T. aestivum* Pavon 76 and USU-Apogee seedlings (*n* = 8 for USU-Apogee and 4 for Pavon 76). Error bar indicates standard error.

It has been previously shown that hexaploid wheat is more tolerant to salt than the tetraploid cultivar (Munns and Tester, 2008; Yang et al., 2014). This has been attributed – in part – to the HIGH-AFFINITY K^+^ TRANSPORTER 1;5 (HKT1;5) (Yang et al., 2014). Strikingly, cluster I also shows a remarkable difference between the hexaploid cultivars in RS1 levels, which plays a role in salt response in barley and tomato (Nveawiah-Yoho et al., 2013; Witzel et al., 2014). There might thus also be a salt response difference between *T. aestivum* Pavon 76 and USU-Apogee. We therefore tested this hypothesis in view of the physiological response of seedlings from the two cultivars under salt stress. *T. aestivum* Pavon 76 and USU-Apogee seedlings were grown in the presence of 100 mM salt, which is considered an intermediate osmotic stress, but for young seedlings this concentration might present an osmotic shock (Shavrukov, 2013). Based on the order of magnitude of the difference in RS1 protein abundance we detected between the cultivars, it was expected that the *T. aestivum* USU-Apogee cultivar would be more tolerant to salt stress, whereas the *T. aestivum* Pavon 76 cultivar would be less tolerant. Indeed, whereas the development of *T. aestivum* USU-Apogee seedlings was rather mildly reduced, salt stress strongly arrested the development of *T. aestivum* Pavon 76 seedlings (**Figure 6A**). Shoot length quantification showed a strong decrease in shoot growth, namely almost 95% in *T. aestivum* Pavon 76 seedlings under salt stress versus the control condition, whereas the *T. aestivum* USU-Apogee seedlings showed a much less severe decrease of just 40% (**Figure 6B**). In conclusion, our comparative proteomics approach and dataset is a powerful tool to predict growth and stress responses of *T. aestivum* cultivars, and for the discovery of genes and proteins associated with these responses.

### Identification of Phosphorylated Proteins

**FIGURE 7:**
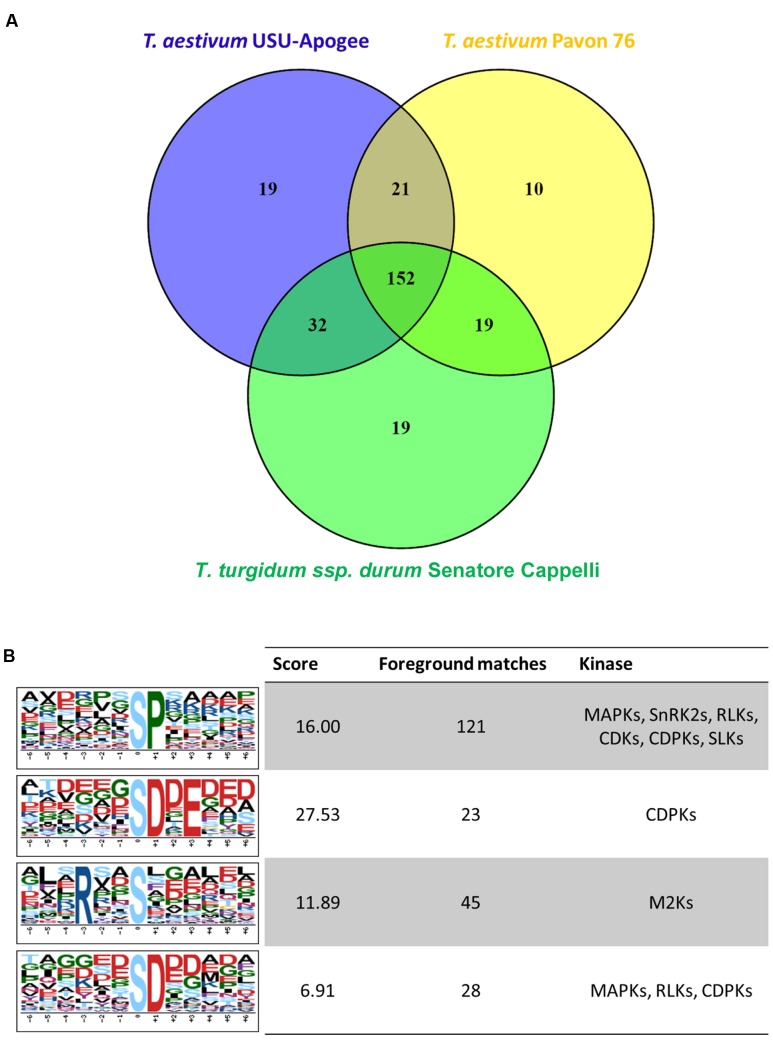
**(A)** Venn diagram representing the numbers of reproducibly quantified phosphosites in each wheat variant. **(B)** Motif-X analysis for amino acid motif enrichment around the identified phosphosites.

Through Ti-IMAC enrichment and subsequent LC-MS/MS analysis, we identified 376 phosphopeptides containing 483 phosphorylated sites, representing 291 phosphoproteins in the samples of all three wheat cultivars (Supplementary Table S4). In general, we detected 85% pS, 13% pT, and 2% pY phosphosites. Compared with another study that was performed on young seedling leaves of two *T. aestivum* cultivars (Lv et al., 2014), there was an overlap of 128 phosphosites, implying that 355 phosphosites were uniquely identified in our experiment (Supplementary Table S5). Filtering for phosphosites with at least 3 intensities out of 6 replicates in at least one of the samples resulted in 289 phosphosites. The overlap of these sites between the three wheat varieties was large with 152 phosphosites common to all three cultivars (**Figure 7A**), and none of the phosphosites were statistically significantly different between the wheat varieties according to a multiple sample test (FDR < 0.05).

To date, many kinase-specific phosphorylation motifs have been identified in plants. We used the identified phosphosites to reveal potential phosphorylation motifs and associated active kinases through a Motif-X analysis (**Figure 7B**). Due to the small number of identified sites, no motif enrichment for phosphothreonine and phosphotyrosine was found. Among the motifs for phosphoserine, the proline-directed motif [sP] was the most enriched. Peptides containing this motif are known as important substrates for MAP-kinases (MAPKs), sucrose non-fermenting1- related protein kinase 2 (SnRK2), receptor-like kinases (RLKs), AGC family protein kinases PKA, PKG and PKC, CDKs (cyclin-dependent kinases), CDPKs (calcium-dependent protein kinases) and SLKs (STE20-like kinases). Furthermore, the common acidic motif [sD] and its submotif [sD.E], which are known to be the target of CDPKs among others, were also enriched; whereas the motif [R..s] was the only basic motif found in the analysis.

Recently, we developed the PTM Viewer^4^ (Vu et al., 2016) which we keep up to date and which can be used to query PTMs in plants. The phosphorylated proteins identified in wheat in this work have been added to that database and are publically available. As such, this is the first repository for identified phosphorylated wheat proteins.

## Conclusion

The objective of this research was to perform proteome analysis in wheat and to estimate the differences between *T. aestivum* and *T. turgidum* ssp. *durum* at the proteome level. Furthermore, we also aimed to apply our phosphoproteomics pipeline to report a number of phosphosites, some uniquely identified in this study, which now can be consulted in the plant PTM Viewer. Using a straightforward and streamlined platform that was previously adapted for quantitative (phospho)proteomics in *Arabidopsis* and maize, we identified 4450 proteins by shotgun proteomics and 483 phosphosites by phosphoproteomics, from which 2449 proteins (51.8%) and 289 phosphosites (59.8%) allowed for quantitative analysis. In addition, our results suggested a large overlap between the wheat cultivars with respect to detectable (phosphorylated) proteins, suggesting research-focused discovery does not necessarily have to be on the economically most relevant wheat variety. Furthermore, our shotgun proteomics allowed identifying putative growth predictors in wheat and/or likely candidates explaining differential responses to environmental triggers. Finally, our dataset comparing tetraploid and hexaploid wheat proteomes is the starting point to look at genome interactions in protein expression.

## Author Contributions

LV, ES, and IV performed the research, MV and FC developed the database, LV, ID and KG wrote the manuscript, and all authors commented on the text.

## Conflict of Interest Statement

The authors declare that the research was conducted in the absence of any commercial or financial relationships that could be construed as a potential conflict of interest.

## Footnotes

1 http://www.wheatgenome.org/

2 bioinformatics.psb.ugent.be/webtools/ptm_viewer/

3 http://bioinformatics.psb.ugent.be/plaza/versions/plaza_v3_monocots/

4 bioinformatics.psb.ugent.be/webtools/ptm_viewer/
